# The vaginal microbiota, human papillomavirus infection and cervical intraepithelial neoplasia: what do we know and where are we going next?

**DOI:** 10.1186/s40168-016-0203-0

**Published:** 2016-11-01

**Authors:** Anita Mitra, David A. MacIntyre, Julian R. Marchesi, Yun S. Lee, Phillip R. Bennett, Maria Kyrgiou

**Affiliations:** 1 Institute of Reproductive and Developmental Biology, Hammersmith Hospital Campus, Department of Surgery and Cancer, Imperial College London, Du Cane Road, W120NN London, UK; 2 Department of Obstetrics and Gynaecology, Queen Charlotte’s and Chelsea–Hammersmith Hospital, Imperial Healthcare NHS Trust, London, UK; 3 School of Biosciences, Cardiff University, Cardiff, UK; 4 Division of Digestive Diseases, St. Mary’s Hospital, Imperial College London, South Wharf Road, London, UK; 5 Centre for Digestive and Gut Health, Imperial College London, London, UK

**Keywords:** Human papillomavirus, HPV, Cervical intraepithelial neoplasia, CIN, Cervical cancer, Vaginal microbiota, *Lactobacillus* species, Prebiotics, Probiotics

## Abstract

The vaginal microbiota plays a significant role in health and disease of the female reproductive tract. Next-generation sequencing techniques based upon the analysis of bacterial 16S rRNA genes permit in-depth study of vaginal microbial community structure to a level of detail not possible with standard culture-based microbiological techniques. The human papillomavirus (HPV) causes both cervical intraepithelial neoplasia (CIN) and cervical cancer. Although the virus is highly prevalent, only a small number of women have a persistent HPV infection and subsequently develop clinically significant disease. There is emerging evidence which leads us to conclude that increased diversity of vaginal microbiota combined with reduced relative abundance of *Lactobacillus* spp. is involved in HPV acquisition and persistence and the development of cervical precancer and cancer. In this review, we summarise the current literature and discuss potential mechanisms for the involvement of vaginal microbiota in the evolution of CIN and cervical cancer. The concept of manipulation of vaginal bacterial communities using pre- and probiotics is also discussed as an exciting prospect for the field of cervical pathology.

## Background

Cervical cancer, the commonest infection-associated neoplasm, and its premalignant precursor cervical intraepithelial neoplasia (CIN), are caused by strains of the human papillomavirus (HPV). Over 100 subtypes of HPV exist with 13 being identified as high risk (high-risk HPV; hrHPV) and causal of cervical cancer in 100 % of cases [1]. HPVs-16 and -18 are the most oncogenic and prevalent of these and are responsible for around 70 % of cases [2]. The peak age for infection in girls is around 20 years. It is estimated that 80 % of sexually active women will have been infected at some point by age 50 [3]. Over 90 % of HPV infections are transient, being cleared by an incompletely understood immune response within 6–18 months [4], although re-infection with the same or different HPV subtypes can occur [5]. Persistence of the virus is essential for development of high-grade CIN and cervical cancer and factors that correlate with higher persistence rates include age, immunodeficiency, smoking, oral contraceptives and *Chlamydia trachomatis* infection. Emerging evidence indicates that cervicovaginal microbiota plays a substantial role in the persistence or regression of the virus and subsequent disease. This review will summarise this evidence, discuss possible mechanisms linking vaginal bacteria with cervical pathology and finally consider the potential for future therapeutic strategies.

Bacteria account for 50 % of the cells of the human body, and together with archaea and lower eukaryotes are collectively termed ‘human microbiota’ [6, 7]. Due to the limitations of culture-based techniques [8], the composition of microbiota in different body compartments is increasingly studied through the use of next-generation sequencing (NGS) techniques. This may involve shotgun metagenomic sequencing of all of the DNA in a biological samples (human and bacterial) but most commonly involves amplifying, sequencing and analysing specific regions of bacterial 16S rRNA genes, although other rRNA genes (18S for eukaryotic microbes) or genomic regions (for viruses) can be used. A variety of bioinformatics tools and platforms are used to assign resulting sequences to specific microbial taxa at different taxonomic levels as well as for in-depth phylogenetic analysis of microbial community structure. There are several excellent reviews covering these topics in greater depth [6, 9, 10], which is beyond the scope of this review.

The link between health, disease and the human microbiota is a fast-moving and contentious area of research, and an appreciation of the variation in microbiota composition amongst individuals is expanding our understanding of the pathophysiology underlying a variety of diseases affecting many body systems, from colorectal cancer [11, 12] to atopic dermatitis [13]. In the majority of human body sites to be examined to date, highly diverse microbial communities are generally considered a signature of health [14, 15]. However, in the case of the female reproductive tract, health is more commonly associated with low microbial diversity and dominance by only one or a few species of *Lactobacillus* [16–18]. Ravel and co-workers demonstrated that this concept is broadly observed in the majority of reproductive age women; a significant proportion harbour comparatively diverse vaginal bacterial communities [17]. In their study, vaginal samples collected from 396 ethnically diverse reproductive age women were analysed using Roche 454 FLX-based sequencing of bacterial 16S rRNA genes. In total, 282 taxa were identified and, using hierarchical taxonomic clustering, the vaginal microbial profile of each woman could be classified into a total of five ‘community state types’ (CSTs), which have subsequently been used by numerous other studies [16, 19, 20]. CSTs I, II, III and V are characterised by dominance of *Lactobacillus crispatus*, *L. gasseri*, *L. iners* and *L. jensenii* respectively and tend to have low species diversity and evenness. In contrast, CST IV is typically devoid of *Lactobacillus* spp. and instead enriched with strict anaerobic species often associated with bacterial vaginosis (BV) including *Gardnerella*, *Megasphera*, *Sneathia* and *Prevotella*. As will be discussed below, the structure of the vaginal microbiota (VMB) is dynamic and transitioning between CSTs can occur. In the vagina, the most common transition observed is from CST III to CST IV [21], which suggests that *L. iners* may be less able to inhibit colonisation of strict anaerobes and pathobionts compared to other *Lactobacillus* spp. [21] or because *L. iners* appears more capable of surviving and adapting to a wide range of pH and other metabolic stress-related conditions due to the constitutive and inducible expression of genes not seen in other lactobacilli [22, 23].

BV is a polymicrobial disorder characterised by a microbial community structure consistent with CST IV; that is, by diminished levels of *Lactobacillus* spp. with associated overgrowth of anaerobes, particularly *Gardnerella*, *Prevotella* and *Peptostreptococcus* species [24]. The prevalence of BV is around 9 % in the UK [25] and up to 29 % in the US [26]. The Hay/Ison criteria used for diagnosis of BV in the UK is largely based on the presence or absence of both *Lactobacillus* and *Gardnerella* or *Mobiluncus* morphotypes [27]. BV has previously been correlated with higher incidence, prevalence and persistence of HPV infection and with development of CIN [28–31]. However, other studies did not find a positive correlation between CIN and BV [32–34], which may partly be explained by the lack of objective diagnostic tests for BV, the reliance on subjective scoring systems [35] and the heterogeneity of BV itself.

### Factors influencing vaginal microbiota composition

The composition of vaginal microbiota is influenced by numerous factors. Ethnicity is a major intrinsic factor known to be significantly associated with variance in community composition, with Caucasian and Asian women displaying a significantly greater prevalence of *Lactobacillus* spp. dominant microbiota, compared to Hispanic and Black women [17]. These differences may be due to genetic factors that influence mucosal immunity or metabolic pathways, which result in preferential conditions for particular species, and could also be due to variation in differing hygiene practices. Menstrual hygiene practices are significantly influenced by cultural and social factors [36], and vaginal douching, discussed below, was reported by 22 % of the 3739 American women sampled in a large representative cohort [37], and is twice as common in Black women, compared to Caucasians [38]. Female hormones also have a major impact on both the structure and stability of vaginal microbial communities. While the human vagina is thought to be initially sterile at birth, rapid colonisation with *Lactobacillus* spp. occurs supported by maternal oestrogen. Reduced oestrogen levels 3–4 weeks post birth correspond with a reduction in vaginal *Lactobacillus* and increased species diversity with enrichment of strict anaerobe and enteric species, which is maintained until puberty [39]. Increased oestrogen and progesterone secretion preceding menarche drives reduced VMB diversity and increased relative abundance of *Lactobacillus* spp. [40]. Throughout a woman’s reproductive age, fluctuation of VMB composition can be linked to the cyclical secretion of oestrogen and progesterone throughout the menstrual cycle. Highest diversity and instability is observed at the time of menstruation [41, 42] which oestrogen and progesterone levels are at their lowest. The presence of menstrual blood also augments the composition of the vaginal mucosa and surrounding environment, leading to depletion of certain species and enrichment of others. Greatest stability of VMB structure over the menstrual cycle is observed at the time of the oestrogen peak, followed by the progesterone peak a few days later [41]. Following menopause, reduced oestrogen and resulting vaginal atrophy are thought to lead to *Lactobacillus* spp. depletion and increased diversity [19]. Consistent with these findings, we have recently shown that the postpartum period of pregnancy, which involves a 100–1000-fold decrease in circulating oestrogen concentrations, is associated with a significant increase in vaginal microbial diversity and richness [16]. The mechanism by which hormones drive vaginal microbial composition is yet to be fully elucidated, but *Lactobacillus* spp. dominance appears to be strongly influenced by oestrogen-driven maturation of the vaginal epithelium, which leads to the accumulation of glycogen in vaginal epithelia [43]. Host α-amylase, present in vaginal mucosa, metabolises the glycogen to simple sugar products such as maltose, maltotriose and maltotetraose that appear to preferentially support *Lactobacillus* spp. colonisation [44].

The widespread use of synthetic hormones for contraceptive purposes also has an impact on the composition of vaginal microbiota. Meta-analysis has shown that hormonal contraceptive use is associated with a 31 and 32 % reduction in recurrent and prevalent BV and 18 % reduced risk of incidence [45]. The study included both combined hormonal contraceptives (combined oral contraceptive pills and vaginal NuvaRing®) and progesterone-only hormonal contraceptives (progesterone-only pills, depot medroxyprogesterone acetate, Mirena® intrauterine devices and implants). Neither combined nor progesterone-only preparations were shown to be more protective than the other. Besides the use of hormonal contraceptives, other environmental factors known to influence VMB composition include smoking [46] and recent intercourse [47], both of which are associated with reduced relative abundance of *L. crispatus* and increase species diversity. Vaginal douching, particularly after menstruation, has been shown to significantly increase the risk of BV [48], and cessation of the practice may reduce the risk of BV [49]. A recent study of 1271 American women has also shown douching to increase the risk of HPV infection with high-risk subtypes in particular [50]. Interestingly, the authors of a meta-analysis concluded that douching may increase the risk of CIN and cervical cancer [51], which may be due to the process resulting in an increased bacterial diversity, which as discussed above is associated with cervical disease.

### Vaginal microbiota and HPV/CIN/cervical cancer

Lee and colleagues were the first to use NGS to examine the impact of HPV infection on VMB composition in a cross-sectional cohort of 912 women participating in a Korean twin study, which included 16 premenopausal, monozygotic twin pairs, 9 of whom were HPV discordant. In the latter group, the investigators observed a profound difference in the VMB structure between twins, with HPV-positive women having higher species diversity and significantly less *Lactobacillus* spp. presence compared to their uninfected twin [52]. Furthermore, they identified *Sneathia* spp. to be a microbiological marker of HPV infection (Table 1).

**Table 1:** Characteristics of studies exploring the association of HPV infection and cervical preinvasive and invasive cervical disease to the vaginal microbiome using next-generation sequence techniques

Study	Summary of findings	Study characteristics
Lee et al. [52]	Summary of findings − HPV positivity = higher diversity and lower proportion of *Lactobacillus* spp. compared to HPV-negative women (19 HPV-positive women vs 26 HPV-negative women) − *Sneathia* spp. = microbiological marker of HPV infection (19 HPV-positive women vs 26 HPV-negative women) − *L. iners* reduced in HPV-positive vs negative monozygotic (MZ) HPV-discordant twins (9 twin pairs, 18 women) (*P* = 0.03)	Participants: 912 women who participated in the Healthy Twin Study, a part of the Korean Genome Epidemiology Study; 68 female twins, their mothers and sisters including 9 HPV infection-discordant MZ twin pairs without CIN and 45 premenopausal women with or without HPV infection Sexual history meta-data: not reported VMB sampling: clinician-collected high vaginal swabs HPV testing technique: MY09/MY11 and GP5+/GP6+, PCR amplicons of 450 and 150 bp and HPV typing (high vs low risk) NGS technique: 16 s rRNA gene regions: V2 and V3, primers barcoded: 8F and 534R, platform: Roche 454 Life Sciences FLX Titanium
Brotman et al. [19]	− CST was significantly associated with remission of HPV (*P* = 0.008) − CST IV-A higher transition to HPV positivity compared to CST I (aTRR: 1.86, 95 %CI 0.52–6.74) − Fastest remission of HPV infection - CST II (aTRR: 4.43, 95 % CI 1.11–17.7 when compared to CST I) − Slowest remission of HPV infection = CST IV-B (aTRR: 0.33, 95 % CI 0.12–1.19 when compared to CST I)	Participants: premenopausal women taking bi-weekly samples over 16-week period as part of a douching cessation study; 5 consistently HPV negative, 2 positive for 1 HPV subtype, 25 positive for 2 or more HPV subtypes Sexual history meta-data: monogamous relationship, number of lifetime sexual partners and daily diary including frequency and type of intercourse and type of contraception used VMB sampling: mid-vaginal swabs, self-sampling HPV testing technique: Roche Linear Array HPV Genotyping Test (37 high- and low-risk subtypes) NGS technique: 16 s rRNA gene regions: V1–V2, primers barcoded: 27F and 338R, platform: Roche 454 Life Sciences FLX Titanium machine
Mitra et al. [54]	− CST IV associated with increasing disease severity (normal = 10 %; LSIL = 21 %; HSIL = 27 %; ICC = 40 %) − CST I negatively associated with increasing disease severity (normal = 50 %; LSIL = 42 %; HSIL = 40 %; ICC = 20 %) - higher levels of *S. sanguinegens* (*P* < 0.01), *A. tetradius* (*P* < 0.05), *P. anaerobius* (*P* < 0.05) associated with HSIL vs LSIL − Lower levels *L. jensenii* (*P* < 0.01) associated with HSIL vs LSIL	Participants: 169 premenopausal women attending colposcopy clinic; 20 normal, 52 LSIL, 92 HSIL, 5 ICC Sexual history meta-data: history of intercourse in 48 h prior to sampling, type of contraception used VMB sampling: clinician-collected, high vaginal swab HPV testing technique: Abbott RealTime HR HPV assay (Abbott M2000 platform) NGS technique: 16 s rRNA gene regions: V1-V2, primers barcoded: 27F and 338R, platform: Ilumina MiSeq
Oh et al. [56]	Higher risk of CIN for the higher vs the lower tertile of − *A. vaginae*, *G. vaginalis*, *L. iners* predominance with a minority of *L. crispatus*: OR 5.80, 95 % CI 1.73–19.4 - *A. vaginae*: OR 6.63, 95 % CI 1.61–27.2 − Risky microbial pattern in presence of oncogenic HPV: OR 34.1, 95 % CI 4.95–284.5	Participants: 120 premenopausal women attending gynaecological oncology clinics; 70 CIN cases: CIN1 (*n* = 55), CIN2 or CIN3 (*n* = 15), controls: normal cytology (*n* = 25), ASCUS (*n* = 25) Sexual history meta-data: not reported, use of oral contraception recorded VMB sampling: clinician-collected digene cervical sampler brush HPV testing technique: hybrid capture II DNA Test (Qiagen, Gaithersburg, MD, USA) NGS technique: 16 s rRNA gene regions: V1–V3, primers barcoded: not stated, platform: Roche/454 Genome Sequencer Junior
Piyathilake et al. [57]	Summary of findings − *L. iners* and unclassified *Lactobacillus* spp. associated with higher CIN2+ rates compared to diverse taxa unclassified *Lactobacillus* spp, *L. iners*, Bifidobacteriaceae, Clostridiales, Allobaculum (OR = 3.48, 95 % CI 1.27–9.55) − Lactobacillaceae, Lactobacillus, *L. reuteri* and several sub-genus level Lactobacillus OTUs higher in women with CIN2+ vs CIN1 − DNA oxidative damage does not correlate with VM structure	Participants: 430 hrHPV positive women aged 19–50 years attending colposcopy clinics; 340 cases: CIN2 (*n* = 208), CIN3 (*n* = 132), 90 non-cases: all CIN1 Sexual history meta-data: not reported, use of oral contraception recorded VMB sampling: clinician-collected high vaginal swabs (Merocel ophthalmic sponges) HPV testing technique: Roche Diagnostics Linear Array NGS technique: 16 s rRNA gene region: V4, primers barcoded: not stated, platform: Illumina MiSeq
Audirac-Chalifour et al. [55]	Summary of findings − VMB diversity significantly higher in CIN and ICC compared to normal, HPV-negative women (*P* = 0.006, *P* = 0.036, respectively) − *L. crispatus* and *L. iners* predominate in normal women − *Sneathia* spp. predominate in women with CIN − *Fusobacterium* spp. in women with ICC − Highest mean levels of IL-4 and TGF-β1 mRNA in *Fusobacterium* spp. VMBs	Participants: 32 women aged 22–61 years, selected from a biobank, recruited from the gynaecological service at a National Cancer Institute; 20 normal (10 HPV negative, 10 HPV positive), 4 CIN (all HPV positive), 8 ICC (all HPV positive) Sexual history meta-data: age at first intercourse, number of lifetime sexual partners, no sexual activity ‘in previous days of the sampling’ (number of days not stated) VMB sampling: cervical scraping swabs from normal women and fresh cell biopsies from women with CIN and ICC HPV testing technique: Seegene Anyplex II HPV HR Detection assay NGS technique: 16 s rRNA gene regions: V3–V4, primers barcoded 347F and 803R, platform: Roche/454 Genome Sequencer Titanium system

*aTRR* adjusted transition rate ratio, *A. vaginae* Atopobium vaginae, *CI* confidence interval, *CIN* cervical intraepithelial neoplasia, *HPV* human papillomavirus, *hrHPV* high-risk HPV, *HSIL* high-grade squamous intraepithelial lesion, *ICC* invasive cervical cancer, *L* Lactobacillus, *LSIL* low-grade squamous intraepithelial lesion; *MZ* monozygotic twins, *NGS* next-generation sequencing, *OR* odds ratio, *OTUs* operational taxonomic units, *SIL* squamous intraepithelial lesion, *VM* vaginal microbiome

In a longitudinal study, Brotman and co-workers studied a North American cohort of 32 sexually active, premenopausal women over the course of 16 weeks using self-sampling at twice-weekly intervals [53]. From a total of 930 samples, women with CSTs III and IV were most likely to be HPV positive (71 and 72 %, respectively) (Table 1). In addition to examining the link between the vaginal microbiota and HPV acquisition and persistence, Brotman and colleagues also suggested that CST II, dominated by *L. gasseri*, may be associated with the most rapid clearance of acute HPV infection. The authors defined rapid clearance as transition from HPV negativity to positivity, and back to negativity, and used continuous time multi-state Markov modelling to calculate adjusted transition rate ratios. Such an observation might point to *L. gasseri* as a potential therapeutic species for maintaining cervical health; however, it is pertinent to note that only two of the 32 women had a predominantly CST II VMB, and that two additional women in the study with CST III and CST IV VMBs also exhibited the same rapid patterns for acquisition and clearance over the 16-week study period. Further studies are necessary to confirm temporal relationships between vaginal microbiota and HPV infection and to determine whether any difference exists in the dynamics of high- and low-risk HPV subtypes, which is most clinically relevant.

Several additional cross-sectional studies have recently been undertaken to characterise the VMB in women with cervical lesions. We have recently studied 169 women in the UK (20 normal controls, 52 low-grade squamous intraepithelial lesion (LSIL), 92 high-grade squamous intraepithelial lesion (HSIL) and five invasive cervical cancer (ICC)) and showed that increasing severity of CIN was associated with higher VMB diversity and decreased relative abundance of *Lactobacillus* spp. [54] (Table 1). A step-wise increase in prevalence of CST IV with increasing disease severity was also observed. While the normal healthy controls in this study displayed 10 % prevalence of CST IV, consistent with previous studies of disease-free individuals [17], the prevalence of CST IV was two-, three- and four-fold higher in low-grade CIN, high-grade CIN and invasive cervical cancers, respectively. In addition, women with high-grade CIN had significantly higher levels of *Sneathia sanguinegens*, *Anaerococcus tetradius* and *Peptostreptococcus anaerobius* and lower levels of *L. jensenii* compared to those with low-grade CIN [54]. A subsequent study of vaginal microbiota and vaginal mucosal cytokine profiles in 32 Mexican women (20 normal, 4 with SIL and 8 with cervical cancer) corroborated some of these findings albeit in a smaller sample size [55]. In this study, increased diversity and a greater relative abundance of *Sneathia* spp. and members of the closely related *Fusobacterium* spp. were shown to be associated with increased disease severity (Table 1). In particular, increased *Fusobacterium* spp. relative abundance was associated with higher levels of IL-4 and TGF-1β mRNA, which the authors suggested may provide local immunosuppression facilitating HPV immune evasion and disease development.

A study in 70 Korean women with CIN and 50 healthy controls used regression modelling and calculated relative excess risk due to interaction and synergy indices to determine biological interactions between vaginal microbiota and hrHPV [56]. Their results were in accordance with our study [54] and Audirac-Chalifour and co-workers [55], concluding that women with CIN had a higher vaginal diversity than healthy controls. The study also identified presence of *Anaerococcus vaginae*, *Garderella vaginalis* and *L. iners* in the absence of *L. crispatus* to be the most high-risk combination for development of CIN, with an odds ratio (OR) of 34.1 for CIN in the presence of hrHPV, compared to hrHPV-negative women. The existence of *A. vaginae* alone was associated with an OR of 29.9 in HPV-positive women, compared to negative women in the cohort. Interestingly, the OR of CIN in hrHPV positive women with *L. iners* was 10.9 (Table 1).

Piyathilake and colleagues [57] have also studied women with CIN (cytologically defined HSIL (*n* = 340) vs LSIL (*n* = 90); all women were hrHPV positive) and used the Dirichlet multinomial mixture model to partition samples into four different metacommunities (partitions 1–4), rather than the previously defined CSTs. Unlike the three previously described studies, this group did not find a high vaginal microbial diversity (CST IV/BV-like VMB) to be associated with HSIL. Partition 3 dominated *L. iners* and unclassified *Lactobacillus* spp. had higher HSIL levels as compared to those with diverse taxa unclassified *Lactobacillus*, *L. iners*, *Bifidobacteriaceae*, *Clostridiales* and *Allobaculum* (partition 1) (OR = 3.48, 95 % CI 1.27–9.55) (Table 1). Such an observation may arise due to ethnic differences between studies. The authors also tested the hypothesis that particular VMB structures may induce oxidative DNA damage, through measuring 8-hydroxy-2′-deoxyguanosine (8-OHdG) levels; a well-characterised biomarker of oxidative stress-induced DNA damage, which has previously been shown to be elevated in SIL compared to healthy controls [58]; however, they did not find a significant correlation.

All four studies in patients with CIN [54–57] are observational studies, and with lack of longitudinal data, it is only possible to demonstrate association with disease states rather than causality. This has been acknowledged as one of the current limitations of ongoing research into the ‘oncobiome’; that is the microbiota associated with cancer development [59]. Although Brotman and colleagues [53] have shown that certain vaginal microbiota may increase a woman’s chance of acquiring transient and persistent HPV infections, there is much work to be done to interrogate the sophisticated relationships between the host, the microbiota and carcinogenesis. However, if a causal link were to be established, the clinical impact would be profound and open up the potential for therapeutic strategies involving the manipulation of the vaginal microbiota away from disease-causing species or structures and towards those associated with protection and health.

## Potential mechanisms of vaginal microbiota-mediated cervical health and disease

Recent observational cross-sectional studies support the concept that CSTs III and IV, in particular, are frequently linked with the presence of HPV infection and development of preinvasive cervical disease states [52–54]. While microbial diversity is considered to be a sign of health in many body sites, highly diverse vaginal microbiota are often considered atypical or a state of dysbiosis and are associated with disease states. However, there is a lack of investigation into how exactly the vaginal microbiota could play a role, and further mechanistic studies are warranted. Vaginal *Lactobacillus* spp. prevent colonisation of bacterial vaginosis-associated bacterial species through maintenance of a low pH [60–63] and bacteriocin production [64–66]. This is important for maintenance of the cervical epithelial barrier function that inhibits entry of HPV to the basal keratinocytes [67]. When BV-associated strict anaerobes are able to colonise, they produce enzymes and metabolites, which may compromise this barrier, facilitating HPV entry. They also act on several cellular pathways that can enable a persistent, productive viral infection and subsequent disease development and progression [68–72]. Indirect evidence generated by existing mechanistic studies in vivo and in vitro in complementary fields and models of BV, as well as studies of other viral genital infections, supports potential mechanisms that warrant further investigation.

### Vaginal pH, lactic acid and hydrogen peroxide

The observational studies discussed in this review all point towards presence of specific species of *Lactobacillus* spp. as potential protective factors against acquisition and persistence of HPV and ultimately development and progression of CIN. This genus is well known to express enzymes capable of glycogen fermentation, which is present at high levels in the oestrogenised cervical and vaginal epithelium, thus producing large amounts of lactic acid [73]. As a result, a strong correlation between high *Lactobacillus* spp. relative abundance in the vagina and low pH exists. This acidic environment can inhibit growth of several potentially pathogenic species, such as *Chlamydia trachomatis*, *Neisseria gonorrhoeae* and *Gardnerella vaginalis* [60–63], yet provides optimal support for cellular metabolic function of the cervix and the vagina [74]. In a study of 9165 Costa Rican women, vaginal pH greater than 5 was shown to be significantly associated with a 10–20 % increased risk of HPV positivity in premenopausal women [75]. The HPV E5 protein responsible for viral transformation is known to be particularly susceptible to low pH [76], which is one plausible mechanism for this observation. Although a low pH environment promoted by lactic acid may be considered generally protective, HPV infection and development of CIN may be additionally influenced by the chemical structure of the lactic acid molecule itself. Lactic acid is a chiral compound with a D- and L-isomer, with the former being predominately produced by *L. jensenii*, *L. crispatus* and *L. gasseri*. However, the L-isomer of lactic acid is produced by the vaginal epithelium, *L. iners* and various anaerobes associated with dysbiosis [77]. Women with CSTs III and IV therefore exhibit a higher ratio of L- to D-lactate, which can lead to increased expression of extracellular matrix metalloproteinase inducer (EMMPRIN) and activation of matrix metalloproteinase (MMP-) 8. This expression could feasibly lead to altered cervical integrity [77] and facilitate entry of HPV to the basal keratinocytes, where the virus thrives. Additionally, high concentrations of D-lactate produced by *L. crispatus*-dominant microbiota have been recently shown to increase cervicovaginal mucus viscosity and enhance its viral particle trapping potential [78].

Previous studies have shown higher rates of bacterial vaginosis in women with lower vaginal levels of hydrogen peroxide (H_2_O_2_) producing bacteria [79]. Unlike the majority of *Lactobacillus* spp., *L. iners* is unable to produce H_2_O_2_, which has also been shown to have antibacterial and antiviral properties [80–82]. However, further studies have shown that under the hypoxic conditions of the vagina, bacteria are unable to make significant levels of H_2_O_2_, which is subsequently present at low levels in the human vagina, and these physiological levels are unable to inhibit growth of BV-associated species in vitro [83, 84]. The observation that *L. iners* often predominates in the presence of HPV infection [53] and CIN [56, 57] may also be linked to the relative instability of this CST in comparison to other *Lactobacillus* spp.-dominant CSTs [21], allowing growth of strict anaerobes resulting in transition to CST IV, which as previously discussed is commonly found in association with dysplasia [54–56]. However, vaginal lactobacilli can exhibit cytotoxic effects on cervical tumour cells in vitro, independent of pH and lactic acid, without the same effects on normal cervical cells [85]. This data suggests that *L. iners* has many properties that differ from most other *Lactobacillus* spp., which could explain why this species does not prevent strict anaerobic growth well, and suggests additional or alternative protective mechanisms against dysplasia on the part of other *Lactobacillus* spp.

*L. crispatus* is infrequently found in coexistence with other bacterial species, tending to be either strongly dominant, or absent, and is the least likely to transition into CST IV [41]. This led us to conclude that the species is strongly resistant to co-colonisation of other bacteria, and thus its presence is consistently associated with health. Women with this microbiota structure not only have the lowest pH of all five CST’s [17], they are also less likely to be infected with bacterial sexually transmitted infections (STIs), Herpes simplex virus (HSV)-2 and HIV, as well HPV [86]. It is thus unsurprising that the presence of *L. crispatus* (CST I) is negatively correlated with CIN [56].

### Bacteriocin production

Besides influencing pH, as discussed above, species that are protective may inhibit pathogen growth through expression of bacteriocidal and bacteriostatic proteins such as bacteriocins [64] which are through to have evolved as a result of ecosystem competition. Gasserin is an example of such a bacteriocin with Gram-negative and -positive activity that was first isolated from *L. gasseri* but has since been found to be produced by other strains of *L. crispatus* and *L. reuteri*. [64–66]. Biosurfactants are another group of peptides excreted by bacteria that can alter surface tension and thus bacterial adhesion thereby preventing formation of biofilms, which are associated with overgrowth of pathogenic anaerobes, in particular *G. vaginalis* [87]. Recently, strains of *L. crispatus* have been shown to excrete *Lactobacillus* epithelium adhesin (LEA), a compound that mediates adhesion to the intestinal and genital mucosa, but additionally inhibits pilus-mediated adhesion of *G. vaginalis* [88]. There is a notable lack of published evidence to suggest that *L. iners* produces many of the protective peptides mentioned above. Lack of protective peptides may account for the comparatively high rates of transition observed between *L. iners*-dominant microbial communities and CST IV [41]. Microbially produced bacteriocins and biosurfactants may also interrupt viral infiltration [89, 90], but further work is required to understand mechanisms and the relevance to HPV infection.

### Disruption of mucosa and epithelial integrity may aid viral entry

Recently, Borgdorff and colleagues proposed modulation of the vaginal epithelial barrier as an important driver of infection. Using samples derived from a cohort of 50 Rwandan sex workers, they examined changes associated with dysbiosis, which causes increased acquisition of HIV infection, using NGS and a proteomics approaches [67]. Irrespective of HIV status, dysbiosis resulted in disruption of key vaginal epithelial cytoskeletal proteins, with increased cell death, which implies epithelial cell damage and desquamation. This change would potentially facilitate entry of HPV into the basal epithelial cells of the cervical transformation zone where the virus thrives and CIN develops [91]. The next stage in viral persistence is replication and shedding of viral particles. BV is associated with higher shedding of HIV and HSV-2 [92], and *G. vaginalis* in particular has been shown to induce HIV replication in vitro [93]. It is therefore plausible that a similar mechanism may exist for HPV, and that dysbiosis, paucity of *Lactobacillus* spp. or a combination of the two, creates an environment that promotes the viral life cycle, persistence of the infection and ultimately development of dysplasia. BV is commonly diagnosed based on the presence of a characteristic thin, watery foul-smelling vaginal discharge [94], which is thought to arise from squamous cell exfoliation in response to amines produced by particular bacterial species [95] and mucus breakdown. Sialidases are a group of mucin-degrading enzymes produced mainly by *Prevotella* and *Bacteroides* spp. and are found at significantly higher levels in women with BV [96]. Dysbiosis may also result in decreased mucus production [67] and a subsequent decrease viral trapping through antibody capture as well as increased exposure of the cervical epithelium. There also exists evidence that inflammation plays an important role in dysbiosis-induced cervical disease. Clinical studies have shown that levels of vaginal proinflammatory cytokines are higher in women with dysbiosis [69, 70] which can result in chronic inflammation; a well-known factor in carcinogenesis of numerous tissues in the body [97].

### Oxidative stress

Dysbiosis has also been shown to result in higher levels of oxidative stress [68], which can generate reactive oxygen species (ROS) which subsequently cause double-stranded DNA breaks in both the HPV episome and host genome, thus assisting HPV integration and ultimately neoplastic transformation. The HPV E6 oncoprotein [98] is also known to use this mechanism which results in the loss of E1 and E2 genes, and subsequently uncontrolled transcription of E6 and E7 enabling increased cellular proliferation, and decrease apoptosis [99]. Despite this evidence, a recent study by Piyathilake and co-workers [57] did not report any significance associated between VMB composition and oxidative stress-induced DNA damage.

### Cellular targets and a role for specific bacterial species

**Fig. 1:**
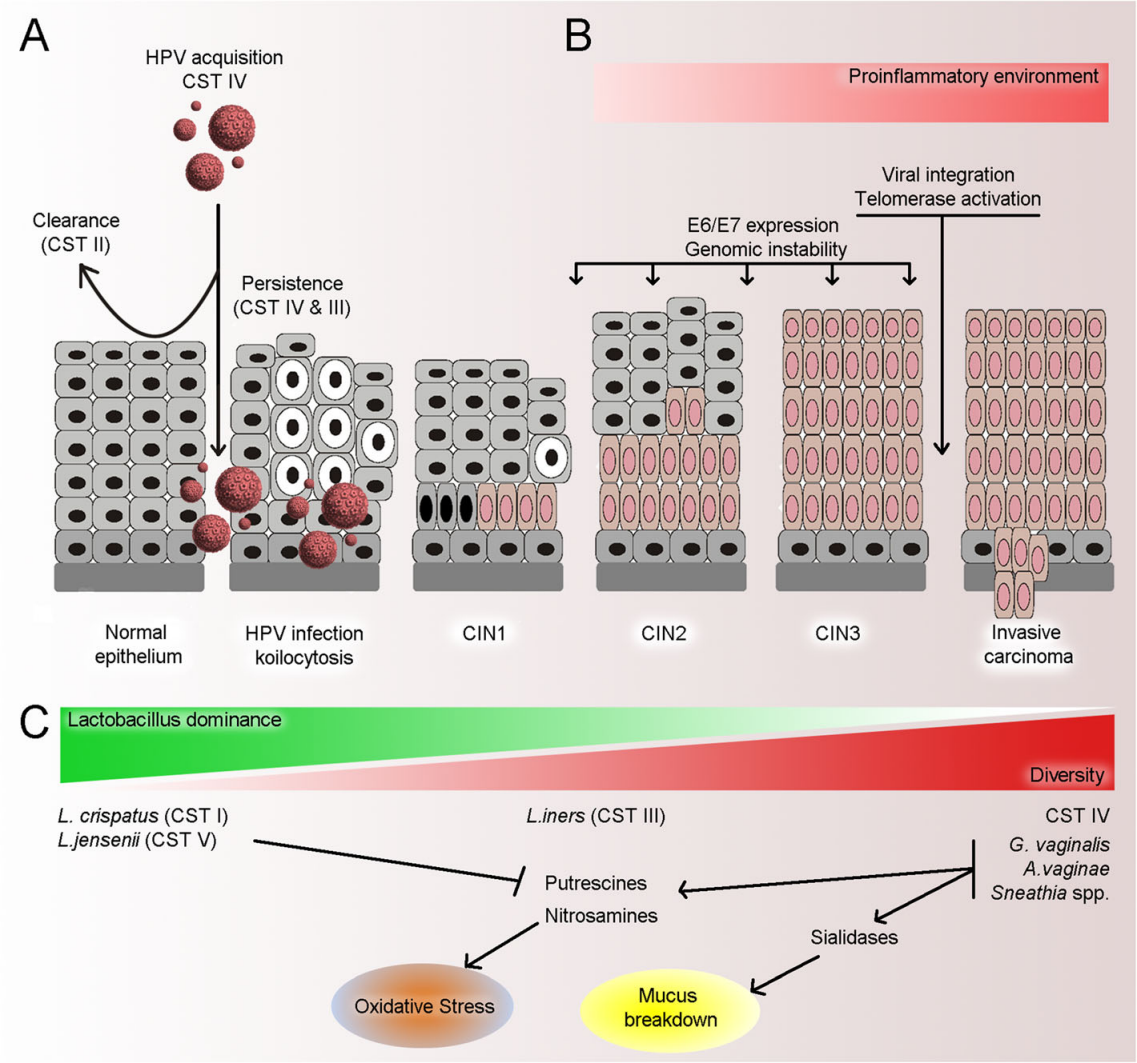
Summary of potential mechanisms associating the VMB with cervical disease. **a** VMB structure appears to be associated with acquisition and persistence of HPV infection, and CST II in particular is associated with most rapid clearance of an acute HPV infection. **b** Dysbiosis can result in a proinflammatory environment, which can facilitate several of the necessary steps in viral transformation including E6 and E7 expression, genomic instability, viral integration and telomerase activation, which are necessary for carcinogenesis. **c** Higher diversity with lower *Lactobacillus* spp. content has been associated with increasing severity of CIN. Particular species associated with high diversity VMBs may produce sialidases which cause mucus breakdown, predisposing the cervical epithelium to tissue damage, as well as producing biological amines which are responsible for oxidative stress; a key mechanism in carcinogenesis. Certain species *Lactobacillus* spp. have been shown to mop up these amines, and therefore their presence may reduce the risk of oxidative damage. *L. iners* does not appear to share many of the protective mechanisms of other *Lactobacillus* species and therefore appears intermediate in its ability to prevent cervical disease

It is currently unclear if dysbiotic vaginal microbial communities act in synergy with HPV to manipulate its cellular targets such as p53, pRB, survivin and hTERT [100] or whether this occurs independently. However, evidence points towards the likelihood that particular species have a pathological role in HPV acquisition and persistence, rather than dysbiosis as a whole. *G. vaginalis* is commonly found in CST IV [17] and often presents at relatively high relative abundance in the adolescent vagina [40]. The immature adolescent cervix is known to be more susceptible to HPV infection compared to older women, and this correlates positively with the rate of squamous metaplasia [101]. It is plausible that the higher levels of *G. vaginalis* may play a role during this period of greater susceptibility. *Sneathia* spp. have frequently been identified in association with HPV positivity [52] and with CIN and cervical cancer [54, 55]. Furthermore, *Sneathia* spp. have also been associated with adverse obstetric outcomes including miscarriage and preterm labour [102, 103]. Previously, Nawrot and colleagues used a PCR-based method to show *Leptotrichia amnionii* (now re-named *Sneathia amnii*) is associated with cervical cancer but not HPV infection or CIN [104]. The species was, however, not unique to women with cancer, suggesting that it may play a role in carcinogenesis, rather than occurring as a consequence of the disease. *Sneathia* spp. belong to the *Fusobacterium* genus, which has further been implicated in colorectal carcinogenesis [12, 105, 106] through activation of proinflammatory pathways and inhibition of immunocytotoxicity [107]. *Fusobacterium* spp. produce FadA, a virulence factor, which is capable of activating the WNT signalling pathway, a key cell survival and proliferation pathway which is found to be dysregulated colorectal carcinogenesis [108] and cervical cancer [71]. Fusobacteria are also implicated in the modulation of immunomodulatory pathways. For example, *F. nucleatum* DNA levels have been shown to be inversely proportional to CD3+-T cell counts in colonic mucosa [109], which is interesting given that aberrant CD3+-T cell signalling and function has been observed in cervical cancers [72] and relapsing disease [110] (see Fig. 1).

## Probiotics, prebiotics and HPV

Probiotics are defined by the World Health Organisation as ‘live microorganisms that, when administered in adequate amounts, confer a health benefit on the host’. They have been used successfully as an adjunct to traditional antibiotics in BV to improve cure rates and prevent recurrence through their ability to replenish the depleted pool of *Lactobacillus* spp. [111].

Metronidazole and clindamycin are the most commonly prescribed antibiotics for BV. They target the overgrowth of anaerobes but do not appear to have a mechanism of action that would actively promote re-establishment of *Lactobacillus spp*. Following successful metronidazole treatment for BV, classified as improvement in Nugent and Amsel’s clinical scoring systems, *L. iners* is often seen to be the predominant re-colonisation species [112], which may arise through expansion of an existing population of this species. An oral preparation of *Lactobacillus rhamnosus* GR-1 in combination with *L. reuteri* RC-14 was shown to increase the prevalence of *Lactobacillus*-dominant vaginal microbiota, as well as improve BV cure rates when given in combination with metronidazole [113]. As neither of these are endogenous vaginal species, the study provides evidence that oral administration of *Lactobacilli* is capable of modulating vaginal microbial structure. These orally administrated bacteria are thought to reach the vagina through a poorly understood mechanism involving transition via the anus and the perineal and vulval skin [114]. A potential mechanism by which these species modulate community structure involves *L. reuteri* secretion of the bacteriocin, Gasserin [66], and *L. rhamnosus* produced Lactocin 160, a similar bacteriocin that is particularly active against *G. vaginalis*. Interestingly, this particular preparation has also been associated with increased relative abundance of *L. iners* as shown using NGS [115]. In a study of bacteria isolated from premenopausal women, PCR-based techniques demonstrated that *L. gasseri* negatively associated with *L. iners and A. vaginae* [116]; two species which co-associate [116] and are suggested to pose an intermediate and high risk for development of CIN respectively [56]. This observation provides further evidence that *L. iners* is not always associated with health, and this should be taken into consideration when designing probiotic therapies, to ensure that they do not promote dominance of this particular *Lactobacillus*. Probiotics have also been suggested as an intervention to promote HPV clearance, and in vitro and in vivo evidence exist to support this technique. Treatment of SiHA cells, an HPV-16-infected cervical cell line, with *Bifidobacterium adolescentis* significantly reduced the production of E6 and E7 mRNAs, suggesting that this species may represent a novel therapeutic of virally transformed cells [117]; however, the efficacy of this species as a probiotic is yet to be proven in humans.

As described above, *L. gasseri* is associated with rapid clearance of incident HPV infections [53]. This species, along with *L. crispatus*, has also been shown to be cytotoxic to HPV-18-infected HeLa cervical cancer cells but not to normal cervical cell lines, independent of pH or lactate concentration, suggesting a more sophisticated mechanism of action [85, 118]. Furthermore, a semi-randomised, interventional study of 54 HPV-positive women with low-grade cervical lesions showed that women treated with oral *L. caseii* showed greater clearance of HPV infections (29 vs 19 %) and were significantly more likely to clear their cervical lesion (60 vs 31 %), compared to an untreated cohort [119].

Prebiotics are indigestible carbohydrates, which include the fructo-oligosaccharide (FOS) and gluco-oligosaccharide (GOS) families, which promote the growth of healthy bacteria already present in the body. They have been most well studied in the gastrointestinal tract, where they have been shown to modulate microbiota composition, as well as exert immunomodulatory effects independent of the microbiota (reviewed in [120]). Several encouraging in vitro studies, and a handful of small in vivo studies, suggest proof of concept in the vagina. FOS and GOS have been shown to promote the growth of *L. crispatus*, *L. jensenii* and *L. vaginalis* in vitro but not *Candida albicans*, *Escherichia coli* or *G. vaginalis*, with the investigators using high-performance liquid chromatography (HPLC) to show that GOS and FOS could not be used as energy sources by the three latter pathobionts [121]. GOS, applied as an intravaginal gel, has been shown in a randomised controlled trial of 42 women, immediately following metronidazole treatment for BV, to result in a significant reduction in Nugent scores at 8 and 16 days of treatment [122]. Konjac glucomannan hydrolysates (GMH) have similarly been shown to promote *Lactobacilli* spp. colonisation in women with *C. albicans* infection [123]. Beyond promotion of bacterial growth through acting as a growth substrate, mannose and GMH have been shown to inhibit adhesion of *E. coli* to human cheek epithelial cells in vitro [124], suggesting additional mechanisms of pathobiont inhibition. When concomitantly administered with probiotics in a synbiotic preparation, they may enhance the growth of probiotic species as well as their bacteriocin production [125]. These results are encouraging and represent a very cheap, safe intervention with few side effects for a disease that cannot otherwise be treated without the risk of significant reproductive and obstetric morbidity [126–130]. While further studies are required to both understand the mechanisms by which the vaginal microbiota plays a role in the pathophysiology of cervical disease, and to identify the most protective species or strain to defend against HPV-induced dysplasia and neoplasia, and their therapeutic doses, pre- and probiotics may offer a practical intervention for the developing world, where cervical cancer is a major cause of female cancer-related mortality [131].

## Limitations of current literature and areas for future study

The ability to derive a causal link between vaginal microbiota and HPV infection and CIN/cervical cancer is limited by the cross-sectional nature of most studies undertaken in this area. This difficulty is further compounded by the slow natural history of the disease, with time from acute HPV infection to high-grade CIN taking years to decades. In addition, numerous other confounders can impact results including smoking [46] and vaginal intercourse without the use of barrier contraception [47], which have both been associated with depletion of *Lactobacillus* spp. The impact of other sexual practices such as oral intercourse, use of lubricants and having multiple sexual partners is poorly understood, and the information gathered in the current observational studies is very heterogeneous as highlighted in Table 1. This information may represent major confounders affecting the VM composition as well as the presence of oncogenic HPV infections and should be clearly documented in future reports.

Furthermore, many of the published studies describe the VM in relatively small cohorts with absent or limited representation of a sizeable group of normal, HPV-negative controls for the described comparisons. Oh and colleagues used samples collected from women with both low-grade and high-grade preinvasive disease and as controls grouped women with normal and ASCUS cytology irrespective of their HPV status [56], including women that may harbour underlying higher grade disease [132]. Piyathilake and colleagues only compared hrHPV-positive patients with CIN1 (non-cases) to those with CIN2 and CIN3 (cases) and lacked a healthy control population [57]. Audirac-Chalifour and colleagues defined as normal controls women with negative cytology and colposcopy, irrespective of HPV status [55]. The study by Mitra and co-workers was the only to group the compared populations according to cytology, histology and HPV status. The authors present subgroup analyses including HPV negative women with normal/ASCUS/LSIL cytology separate to those positive for hrHPV, although the samples in those subgroups were small [54]. Studies must therefore be appropriately designed to permit accurate interpretation of data and ensure any observed changes in vaginal microbial communities are directly associated with the pathology. Appropriately stored samples in historical biobanks are thus a precious commodity, which may present an opportunity to perform longitudinal studies to answer these kinds of questions. There are however limitations as these biobanks often use long interval sampling designs. Prolonged intervals between samples may fail to describe rapid changes in VMB composition occurring throughout the disease process, while important meta-data such as sexual history and smoking may be lacking.

A number of recent studies have led the scientific community to re-think the traditional conceptual model of the natural history of HPV. Previously regarded as a viral infection which simply causes transient infections or persists as a chronic infection based on studies conducted with protracted sampling intervals, it has now been shown that HPV status can fluctuate quickly over a short time period, based on studies involving very frequent testing over a short space of time [53, 133]. Whether this is due to detection and re-detection of low-level persistent infections due to wavering loss and regained immune tolerance, rather than true re-infection, is currently unclear. A small study of HPV status in 20 women has shown that HPV detection peaked at days 7–11 of the menstrual cycle [134], which are the days immediately following the time of highest VM diversity [41]. This suggests that it may be possible to correlate fluctuations in HPV status with the changes in the VM occurring as a result of the menstrual cycle to explain in role of the VM in the emerging, rapidly dynamic model of infection. However, these studies have been performed in normal, presumably healthy women, and to our knowledge the temporal dynamics of HPV status over a short period of time has not yet been studied in women with known CIN. The current theories behind the VM in cervical disease will require re-evaluation if similar HPV dynamics are observed compared to that of healthy women. Furthermore, integration of alternative HPV tests for detecting DNA, such as mRNA and E6/E7 levels, into future microbiome studies may help us begin to answer these important questions. In addition studies, focus on the interplay between the microbiota and the host immune system within these alternative conceptual models on the natural history of HPV infection is also required. It has recently been suggested that the immune system contributes as little as 20 % towards viral clearance, and that stem cell stochasticity plays the biggest role, based on the integration of epidemiological data with mathematical cellular modelling [135]. This model may also explain the concept of latency and thus fluctuating HPV status. How the VM may influence the stochastic dynamics of basal epithelial cells however is unknown.

Alternative approaches for the assessment of vaginal microbiota and its interactions with the host immune system may also offer useful means to monitor HPV acquisition, persistence and subsequent cervical dysplasia and neoplastic transformation. For example, metabonomics, defined as ‘the quantitative measurement of the dynamic multiparametric metabolic response of living systems to pathophysiological stimuli or genetic modification’ [136], is emerging as a novel way to investigate the host-microbe interaction through inspecting functional metabolic changes associated with disease phenotypes [137, 138]. Using nuclear magnetic resonance (NMR) or mass spectroscopy (MS) coupled to separation technologies, e.g. HPLC, it is possible to identify particular metabolites or pathways that are altered in association with the VMB structure. This approach will not only increase our understanding of the impact of bacteria on host biochemical and immune response, which is likely extremely complex [138], but may also present the opportunity for development of novel prognostic tests for triage of patients who are most likely to develop a high-grade or cancerous cervical lesion. The identification of such patients is one of the biggest clinical challenges in current colposcopy practice.

By definition, members of a particular bacterial species have a total nucleotide identity of >70 % across their genomes [139]. The remaining genome diversity gives rise to the existence of different strains, which may have different functional genes that induce different biological properties. Further evidence is required in order to determine whether only certain strains of a particular bacterial species are either protective or pathogenic with regards to HPV and cervical dysplasia, and a recent study by Abdelmaksoud and colleagues [140] hints this is very likely. The team compared strains of *L. crispatus* that colonised women with, and without BV, demonstrated considerable genomic diversity within the species and identified several genes exclusive to the presence or absence of BV [140]. These genes require further investigation to help understand the protective mechanisms exploited by certain bacteria, and other disease-associated species should also be studies using metagenomic techniques, which in turn will support development of appropriate probiotic preparations.

Afro-Caribbean women have a fourfold higher prevalence of CST IV VMBs compared to Caucasian and Asian women [17]. They have also been found to have a higher age standardised rates of cervical cancer (6.3–11.2 per 100,000 women), compared to Caucasian and Asian women (8.2–8.7 and 3.6–6.5 per 100,000, respectively) in the UK [141] and the USA [142]. Although there may be a slightly higher risk of invasive disease, this does not correspond to the increase in the prevalence of CSTIV in black women, suggesting the presence of a far more complex mechanism and interactions between the bacteria and the individual host beyond the simple presence of CST IV that promotes or not HPV persistence and cervical carcinogenesis. Future studies will help to further explore these possible associations in long-term samples and comprehend why particular species or VM CSTs may be associated with health in some but disease in others. Compliance with cervical screening and many behavioural and social factors may also explain the higher risk of invasive disease in this ethnic group.

Pre- and probiotics clearly present an enticing novel therapeutic approach to this disease, because they are cheap, easy to administer, with a low side effect profile, unlike the current gold standard treatment for high-grade CIN, which involves a surgical method that carries significant risk to future reproductive outcomes [126–130]. Furthermore, pre- and probiotic use would have an impact in other areas of women’s health, with dysbiosis being responsible for a two- to fourfold increase in risk of preterm birth [143], increased risk of miscarriage [143] and increased rates of HIV transmission [144], which highlights the importance of investing time and resources into exploring this therapeutic strategy.

## Conclusions

The vaginal microbiota appears to play a role in the acquisition and persistence of HPV in the human vagina and in the subsequent development and progression of CIN. There is a need for further longitudinal studies to prove that these disease outcomes are influenced by VMB composition. This information may present the opportunity for development of novel therapeutic agents in the form of probiotics, to prevent HPV infection, promote its clearance in infected women and negate the risk of cervical dysplasia and future adverse reproductive outcomes that are associated with the current treatment methods [126–128]. Mechanistic studies are required to identify the most protective species. Furthermore, it is possible that only certain strains of a bacterial species are able to protect or promote disease processes.

Alongside the bacterial microbiota, the virome is now a new emerging area of interest. Although we have known for many years that HPV is the aetiological agent in precancerous and cancerous pathologies of the cervix and lower genital tract, other viral genera present in the normal vagina, alongside papillomaviridae, may be involved in disease progression [145]. Furthermore, we are aware of a symbiotic relationship between bacterial and viral communities, which requires further investigation specific to HPV and cervical pathology.

## Abbreviations

BV: Bacterial vaginosis
CIN: Cervical intraepithelial neoplasia
CST: Community state type
HIV: Human immunodeficiency virus
HPV: Human papilloma virus
hrHPV: High-risk human papilloma virus
HSV: Herpes simplex virus
NGS: Next-generation sequencing
OR: Odds ratio
STIs: Sexually transmitted infections
VMB: Vaginal microbiota
